# Setting up a maternal and newborn registry applying electronic platform: an experience from the Bangladesh site of the global network for women’s and children’s health

**DOI:** 10.1186/s12978-020-00993-w

**Published:** 2020-11-30

**Authors:** Sk Masum Billah, Rashidul Haque, Atique Iqbal Chowdhury, Md Shahjahan Siraj, Qazi Sadequr Rahman, Tanvir Hossain, Asraful Alam, Masud Alam, Chelsea Marie, Beth McGrath, Shams El Arifeen, William A. Petri

**Affiliations:** 1 Maternal and Child Health Division, icddr,b, 68 Shahid Tajuddin Ahmed Sarani, Dhaka, 1212 Bangladesh; 2 Faculty of Medicine and Health, Sydney School of Public Health, The University of Sydney, Sydney, New South Wales Australia; 3 Infectious Disease Division, icddr,b, Dhaka, Bangladesh; 4 Infectious Diseases & International Health, University of Virginia, Charlottesville, Virginia USA

**Keywords:** Bangladesh, Maternal and newborn health, Global network, Registry, Electronic data capture

## Abstract

**Background:**

The Global Network for Women’s and Children’s Health Research (Global Network, GN) has established the Maternal Newborn Health Registry (MNHR) to assess MNH outcomes over time. Bangladesh is the newest country in the GN and has implemented a full electronic MNH registry system, from married women surveillance to pregnancy enrollment and subsequent follow ups.

**Method:**

Like other GN sites, the Bangladesh MNHR is a prospective, population-based observational study that tracks pregnancies and MNH outcomes. The MNHR site is in the Ghatail and Kalihati sub-districts of the Tangail district. The study area consists of 12 registry clusters each of ~ 18,000–19,000 population. All pregnant women identified through a two-monthly house-to-house surveillance are enrolled in the registry upon consenting and followed up on scheduled visits until 42 days after pregnancy outcome. A comprehensive automated registry data capture system has been developed that allows for married women surveillance, pregnancy enrollment, and data collection during follow-up visits using a web-linked tablet-PC-based system.

**Result:**

During March–May 2019, a total of 56,064 households located were listed in the Bangladesh MNH registry site. Of the total 221,462 population covered, 49,269 were currently married women in reproductive age (CMWRA). About 13% CMWRA were less susceptible to pregnancy. Large variability was observed in selected contraceptive usage across clusters. Overall, 5% of the listed CMWRAs were reported as currently pregnant.

**Conclusion:**

In comparison to paper-pen capturing system electronic data capturing system (EDC) has advantages of less error-prone data collection, real-time data collection progress monitoring, data quality check and sharing. But the implementation of EDC in a resource-poor setting depends on technical infrastructure, skilled staff, software development, community acceptance and a data security system. Our experience of pregnancy registration, intervention coverage, and outcome tracking provides important contextualized considerations for both design and implementation of individual-level health information capturing and sharing systems.

## Plain English summary

In 2001, Global Network (GN), an international consortium dedicated to improving the health of women and children in resource-limited settings was developed. The GN has established an international multi-site population-based registry to quantify and understand the trends in pregnancy outcomes in defined low-resource geographic areas over time. Data from the registry is also used to guide/plan current and future GN studies as well as assessing the impact of the different interventions on maternal and newborn health.

Bangladesh is the most recent country to join the GN and has implemented a fully electronic registry system for married women surveillance, pregnancy identification and follow up until 42 days after birth. An electronic application is used by community-level registry administrators for enrollment of participants, preparation of follow-up visits plans, recording and transferring/sharing information collected at different follow-up visits. The application also provides real-time connectivity with a data display dashboard for progress monitoring and data use. The electronic registry system is better than paper-based methods for improved data quality and integrity, study productivity, preference of users, and reduced cost.

## Background

In 2001, the National Institute for Child Health and Human Development established the Global Network for Women’s and Children’s Health Research (Global Network, GN) [1], an international consortium dedicated to improving the health of women and children in resource-limited areas. It has a goal to understand the morbidity and mortality surrounding childbirth and to identify the scalable low-cost interventions to improve maternal-child health. The GN has established an international multi-site population-based registry to assess pregnancy outcomes over time. The primary purpose of this prospective, population-based observational maternal newborn health registry (MNHR) is to quantify and understand the trends in pregnancy outcomes in defined low-resource geographic areas over time, in order to provide population-based statistics on stillbirths and neonatal and maternal mortality, including cause of death (COD). In addition, MNHR data will be used to guide/plan current and future GN studies as well as assess the impact of the interventions of GN protocols [2, 3].

Accurate data collection and management is an essential component of the MNHR which enrolls approximately 60,000 participants per year across 8 global sites (6). Bangladesh is the most recent country to join the GN and has implemented a full electronic MNH registry system, from married women surveillance to pregnancy enrollment and follow up. Application of electronic data collection (EDC) is an increasingly common tool for health research, especially in surveillance and registries due to improved data quality and integrity, study productivity, preference of users, and reduced cost relative to paper-based methods. Different GN sites of MNHR have adopted electronic data capturing and recoding systems in addition to existing paper-based data collection at service delivery points and home follow-ups. This article details the process of implementing EDC for the MNHR in a rural site in Bangladesh.

## Methods

The MNHR is a prospective, population-based observational study that tracks pregnancies and outcomes in defined geographic communities. It provides stillbirth, neonatal and maternal mortality rates to inform research. The MNHR has been introduced in 7 low-middle income countries (DRC, Guatemala, India, Pakistan, Bangladesh, Zambia, and Kenya) [3]. The MNHR was initiated in 7 sites between 2008 and 2009. In Bangladesh, the MNHR was initiated in March 2019 and is funded to continue through 2023.

### Study variables

The purpose of this observational study is to quantify and understand the trends in pregnancy outcomes in defined low-resource geographic areas over time in order to provide population-based statistics. The MNHR also includes collection of selected intervention coverage and background characteristics of the enrolled study participants to document the changes over time and their relationship with the observed changes in maternal and newborn outcomes [3]. The key variables included in the registry throughout the pregnancy and newborn period continuum are shown in Table 1 and definitions of the variables are included in the annex to Table 1.

**Table 1:** Variables in MNHR in Bangladesh

Level	Indicators		
**Outcome**	Maternal mortality, neonatal mortality, stillbirth, miscarriage, medical termination of pregnancy, birth weight, cause of death, maternal and newborn complications,		
**Intervention coverage**	**Ante-partum**	**Child-birth**	**Post-partum**
	Antenatal care from medically trained provider, Tetanus toxoid vaccination, iron, vitamin and calcium supplementation, HIV test, urine test for protein, ultrasound	Skilled attendance at delivery, place of delivery, mode of delivery, referral for hospitalization, newborn resuscitation, essential newborn care,	treatment received for maternal and newborn complications, hospitalization
**Background characteristics**	Household demographics, wealth, housing characteristics, water source, sanitation, fuel use, maternal education, height, hemoglobin, weight, parity, pervious pregnancy complications, gestational age		

### Study site

The Bangladesh MNHR site of the global network was established at Ghatail and Kalihati upazilas (sub-districts) of the Tangail district, situated about 90 km north-west of the capital Dhaka. The upazilas (sub-districts) have been chosen purposely for the study based on good transportation linkage with the Tangail district headquarters and no other ongoing maternal surveillance or interventional studies. The Kalihati sub-district consists of 1 municipality and 11 unions (lowest administrative unit) with 301 villages. The Ghatail sub-district consists of 1 municipality, 11 unions of 411 villages. We have purposively selected 9 unions from Kalihati (Bangra, Bir Basunda, Elenga, Kok dahara, Nagbari, Narandia, Paikara, Sahadebpur, Salla) and 3 unions (Anehola, Digalkandi, Jamuria) from Ghatail sub-districts. The Selection criteria for the unions were- 1) good accessibility with the upazila headquarters in Tangail, ii) presence of a functioning Union Health and Family Welfare Centre (providing normal delivery care and outdoor-based maternal and newborn services), iii) more than 25% of the area is not covered either by riverine marsh or forest cover, iv) not bisected or bordered by Jamuna river and vi) at least 25,000 population.

We defined 12 MNHR clusters in the 12 selected unions (one in each union). Municipality areas of the two sub-districts were excluded as the primary intention was to establish the registry site in rural areas only. The Bangladesh MNHR site aims to achieve 3600 births per year from 12 study clusters, 300 births from each cluster. The crude birth rate (CBR) in rural Bangladesh is 20.4 per 1000 population [4]. Each cluster is comprised of ~ 18,000–19,000 population or ~ 4400–4700 households (household size 4.1). All pregnancies from each cluster are enrolled and followed up. The total population of 12 clusters in the Bangladesh MNHR site is ~ 216,000.

The 12 clusters have 12 primary health care centers named Union Health and Family Welfare Centers (UH&FWC). Both of the sub-districts have one 50-bed Upazila Health Complex (first level referral facility) and 24 private clinics/hospitals in total each consisting of 5–15 beds and providing inpatient maternal and child care services.

### Data collection tools and methods

**Fig. 1:**
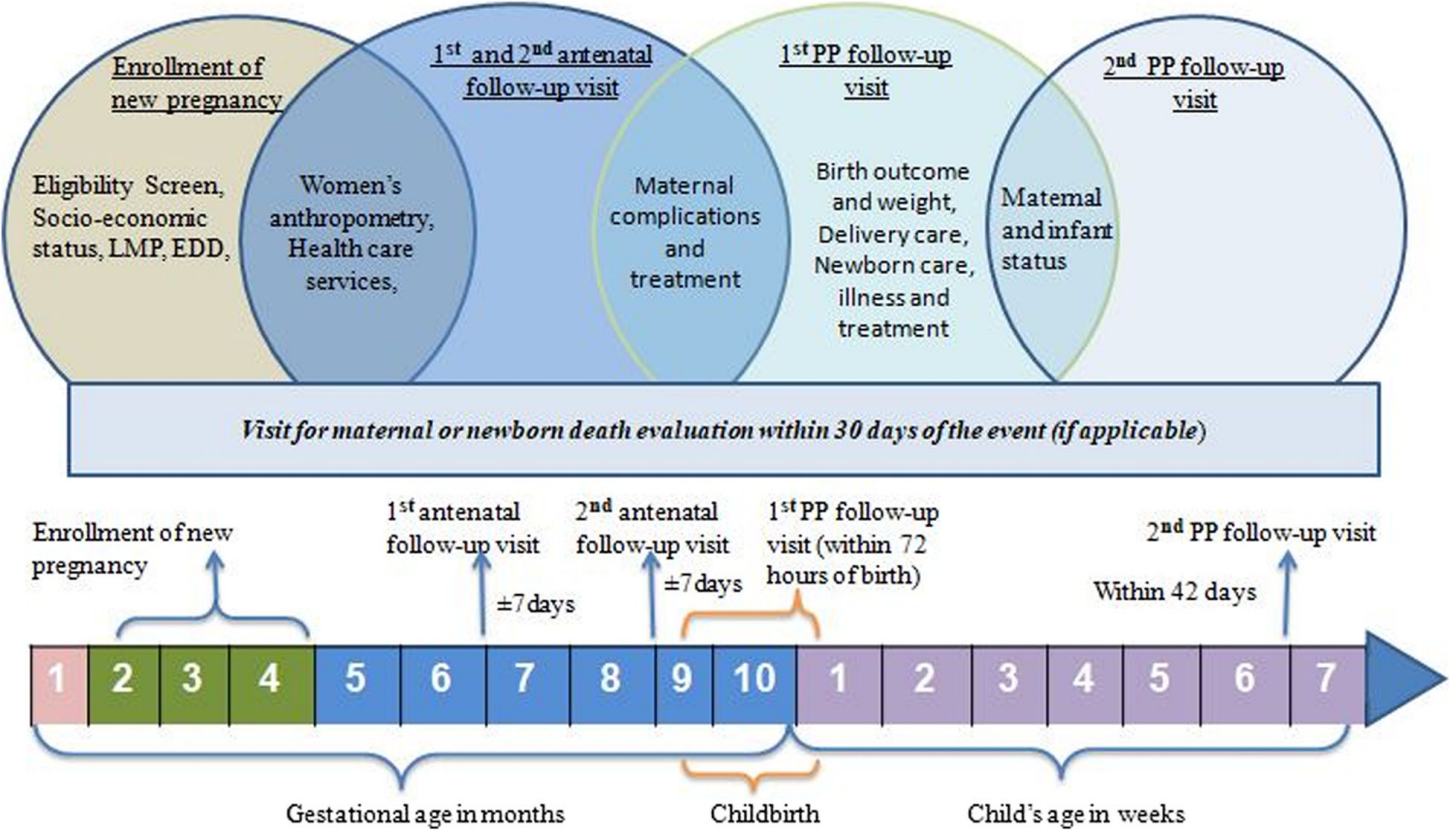
Enrollment and follow up visit schedule and Information collected at different visits

The Bangladesh MNHR has adopted the multi-country registry data collection tools developed by Global Network for Women’s and Children’s health, coordinated by RTI International [3]. The registry aims to enroll women in early pregnancy and collect the primary data outcomes at two time points (within 72 h of birth and 42 days after delivery) for each pregnant woman (Fig. 1). Additionally, the Bangladesh site adds two visits during pregnancy to collect secondary study data and reinforce timely outcome notification by the participants. Similar to several other sites, the Bangladesh site has adopted a house-to-house married women surveillance system and has developed a site-specific married-women surveillance form to identify and enroll pregnancies. Project recruited surveillance workers conduct the married women surveillance on two-monthly rounds. Enrollment and follow up visits are conducted by registry administrators (RAs) at household level for data collection by face-to-face interview of the registry participants. Available prescriptions, case records, mother-child card, discharge certificates are also reviewed (if available) to collect relevant clinical findings and management provided.

Post-partum follow-up visits are also made at health facilities if the participants deliver at health facilities located in the registry sub-districts and remain there during the first 72 h of the post-partum period. However, RAs depend mostly on mother’s recall as the information source as nearly half of the deliveries occur at home. Among the hospital deliveries, the majority occur at several private-for-profit facilities both within and outside of the registry sub-districts with poor and inconsistent record keeping systems. Data on cause of death is collected by trained project research physicians by interview at household level and/or reviewing clinical records within 1 month of a reported maternal, perinatal and neonatal death among the registry participants. A summary of data collection tools, key content, methods and source of information in Bangladesh MNHR site is presented in Table 2.

**Table 2:** Bangladesh MNHR data collection forms, data collection

Data collection tools (forms)	Description	Responsible Person/Method	Potential Source(s) of Data
Married women surveillance form	List of all households, married women and their pregnancy status in the community.	Surveillance workers conduct census of all households in the registry clusters in two monthly rounds.	Household census
Pregnancy Registry Log (MN00):	Registry of all women who are pregnant in the community.	Surveillance workers report identified pregnant women to the Registry Administrator (RA) via electronic record sharing	Household surveillance of married women likely to become pregnant
Enrollment Form (MN01)	Demographics: basic demographic information of women	RAs collect data through interview of the pregnant women identified by surveillance workers	Interview with pregnant women
Perinatal Form (MN02)	Delivery outcome: outcome of all pregnant women on registry and coverage of interventions to be reported at delivery/discharge	RAs collect data through interview with participant, and/or family, reviewing health records at household and/or at health facility during 1st post partum visit	Interview with registry participants and review of health records (where available)
Follow-up (MN03)	Follow-up outcome: status of all mothers (to day 42) and neonates (to day 28) who are on the registry, post-partum complications	RAs collect data through interview with participant, and/or family, reviewing health records at household and/or health facility visits	Interview with mother/mother-in-law, birth attendant, and review of health records
Protocol Deviation (MN04)	Protocol Deviation: used to report all protocol deviations which occur during the registry	RA and the Senior Foreign Investigator (SFI) and/or Study Coordinator or their designee complete after reviewing the report	RA and Study Coordinator
Perinatal Cause of Death (COD) (MN05)	Perinatal COD: used to collect information of all fetal deaths > 20 weeks gestation and neonatal deaths (0 to day 28 days) which occur in the registry	Project Research Physicians collect data and the SFI and/or Study Coordinator or their designee reviews	Interview with mother/mother-in-law/family, birth attendant, and review of health records
Maternal COD (MN06)	Maternal COD: collect data on all maternal deaths for any reason during pregnancy or up to 42 days post-partum to determine a COD	Project Research Physicians collect data and the SFI and/or Study Coordinator or their designee reviews	Interview with family, birth attendant, and review of health records
Ultrasound dating worksheet (MN17)	Used to collect information on date of ultrasound and expected date of delivery	RAs collect the data at enrolment or perinatal (1st post partum) visit if women had received ultrasound any time during pregnancy	Ultrasound report and Interview with the participant
Socioeconomic status variable (MN18)	Used to collect information on household possession of assets, housing characteristics and water and sanitation access	RAs collect the information during enrollment visit at household	Interview with registry participants or other adult household member

### Registry administration process

#### Definition of registry clusters, household listing and creating surveillance blocks

**Fig. 2:**
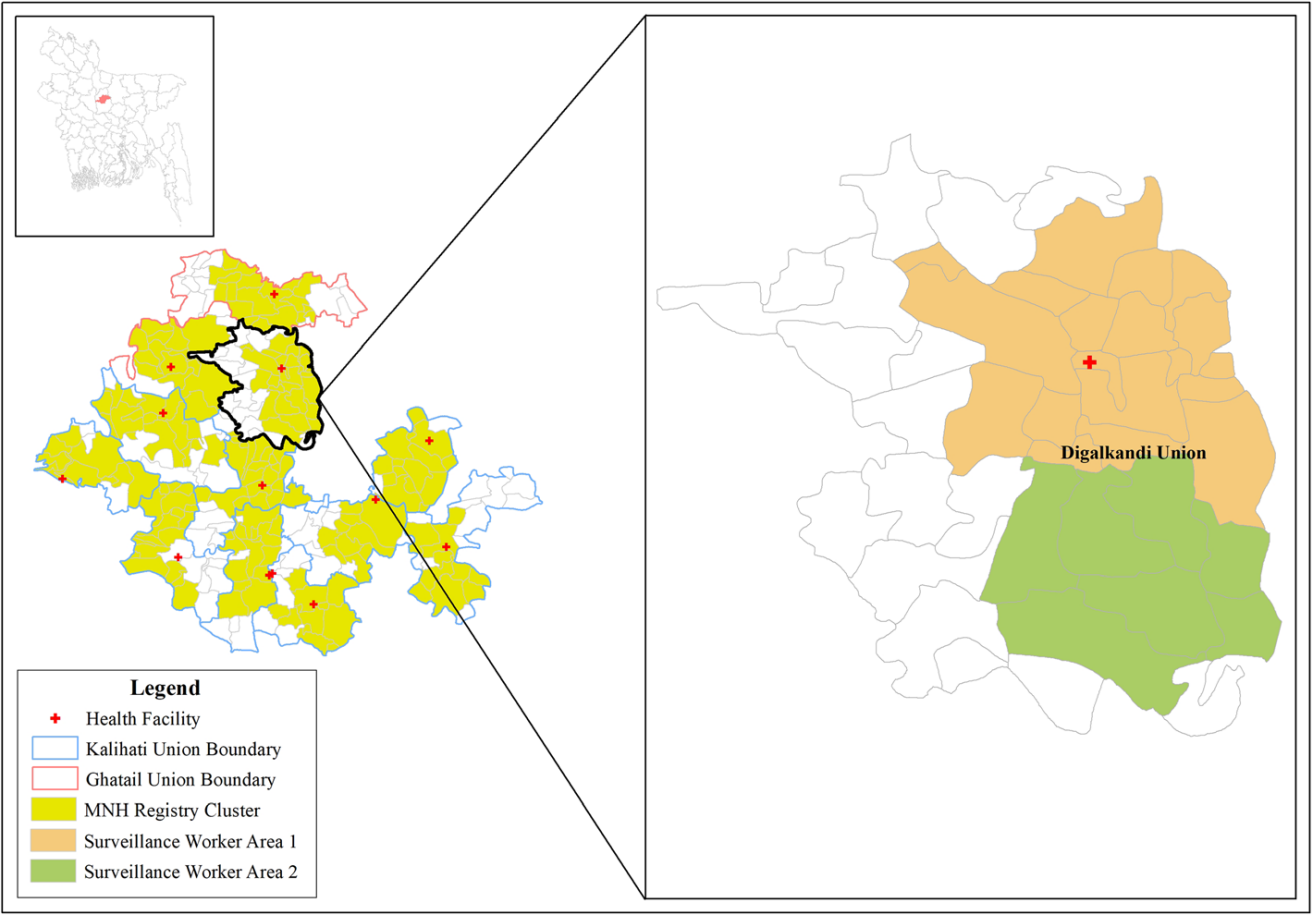
12 clusters in unions (left) and two surveillance workers’ areas (right)

A geographic information systems-based approach was adopted to determine the 12 registry clusters from 12 unions. We collected the cadastral map (map showing land parcels with record numbers) of the unions from the Local Government Engineering Department (LGED). The cadastral map has mouja (lowest revenue unit, becomes synonymous to village) boundaries containing one or more villages. Projected population data from 2011 population census data for villages was linked with the cadastral map. Villages to be included in the registry cluster were selected by desktop-based geographic information system (GIS) exercise. The starting point for village selection was defined by the union level health facility and then expanded until the cumulative population of the catchment reached ~ 18,000–19,000. The cluster boundary was then drawn on a GIS map including the selected villages to define each registry cluster (Fig. 2).

After defining the registry clusters, mapping and listing of all *bari* (homesteads, comprising one or several households with same paternal origins) and households were conducted by a team of 24 project recruited surveillance workers visiting house-to-house. Household listing included recording of household identification and address, number of members and number of married women in reproductive age. Mapping of households included taking geocoordinates of every *bari* and important landmarks of the catchment. If the total population of the initially selected villages (from population census) was not found to be 18,000 after mapping and listing of households the catchment boundary was extended by adding new nearby villages within the unions. The Open Data Kit (ODK) tool, an android based open source application, was used on cell phones to collect geocoordinates of the *baris* and landmarks. The household information was recorded using an electronic application developed for MNH registry on hand-held android-based tablets. Mapping and listing of households will be updated annually.

**Fig. 3:**
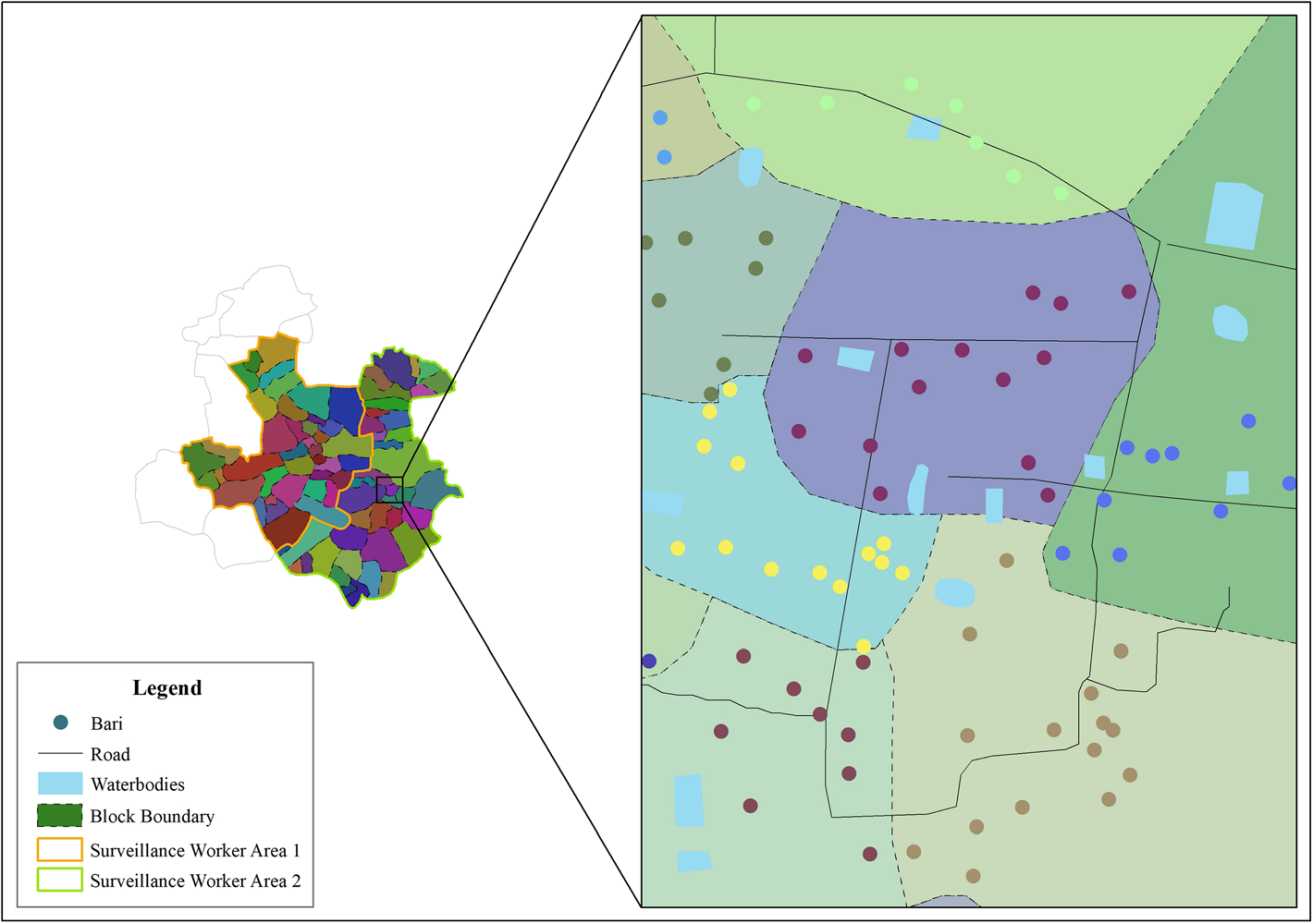
*Baris* in surveillance blocks and surveillance workers’ area in a catchment

The ArcGIS version 10 software was used to process the catchment maps. For preparation of catchment maps, we used high-resolution Google earth pro satellite images as the background. Those images were georeferenced maintaining Root Mean Square Error (RMSE) less than 0.14. After georeferencing, settlement patch, water bodies, and roads were digitized through on-screen digitization process at R.F. scale of 1:3000 to produce geographic features. Coordinates of *baris* and landmark features collected by the ODK system in household mapping overlaid on the digitized geographic features to prepare the final catchment maps showing topographic features and listed households (Fig. 3).

After the cluster mapping and household listing was completed the clusters were divided into 80 blocks, each consisting of 55–65 households. The size of the block was determined by considering the number of households one surveillance workers can feasibly visit in 1 day. Block boundaries were defined by adjoining household locations within the blocks. Then 80 blocks were distributed to two surveillances workers (40 blocks to each) assigned for each catchment. In total, the Bangladesh MNHR site consists of 12 clusters of 960 blocks.

#### Married women surveillance

MNHR Project surveillance workers visit all households in MNHR clusters every 2 months for currently married women surveillance and identification of new pregnancies. As pregnancy outside marriage is socially unaccepted and is a taboo in Bangladesh, unmarried women in reproductive age are not included in the registry. They use a custom-designed married women surveillance application on hand-held tablet computers during the household visits. During household visits, they update the list of existing currently married women of reproductive age, 15–49 years (CMWRA), and track migration in and out of the study cluster. From every listed CMWRA, surveillance workers collect information on date of birth (once only), current contraceptive use, reported pregnancy status, first date of last menstrual period and if the woman had stopped menstruating for more than year or if a hysterectomy was performed. Women with permanent sterility, in menopause, or who have undergone hysterectomy are dropped from the surveillance. Women using long acting methods of contraception are inactivated for the next year due to low chance of becoming pregnant. Women who are susceptible to get pregnant and have passed 45 days or more since the last LMP date are identified as a “possible pregnancy”. The women are invited to participate in a dip-strip pregnancy test performed by the surveillance worker. The women with positive pregnancy results in the dip-strip test are reported to the registry administrator for enrollment in the registry. The registry aims to enroll women early in pregnancy. In subsequent rounds, all new pregnancies were identified by the surveillance workers following the same process and were notified for enrollment.

Surveillance workers are all female with a Higher Secondary School Certificate and are recruited from the local community. Their task of house-to-house surveillance is supervised and monitored by Field Supervisors (FS) and coordinated by a Field Research Supervisor. The surveillance team members were trained extensively on a household listing tool including the provision for listing of all married women of reproductive age and recording their date of LMP. During training, special emphasis was given on different techniques for recalling LMP date as a good proportion of the women may not be able to recall their LMP. Fortnightly meetings are conducted for performance review, field-related troubleshooting and providing feedback.

#### Enrollment and follow-up of registry participants

Pregnant women identified in surveillance are enrolled in the registry. The eligibility criteria for enrollment are given in Table 3.

**Table 3:** Eligibility criteria for enrollment of participants in MNH registry

**Inclusion Criteria**	
• Married pregnant women aged 15 to 49 years	
• Permanent residents of the study cluster including woman intending to transfer for care at delivery (natal move out)	
**Exclusion criteria**	
• Woman not a permanent resident of the study cluster but delivers in the study cluster (natal move in)	
• Women who do not provide consent or assent (for women aged < 18 years)	

Like the other GN registry sites, the Bangladesh site enrolls eligible pregnant women irrespective of their intended place of delivery. The majority of the pregnant women in Bangladesh are unsure of their intended delivery place at the early stage of pregnancy suggesting limited birth preparedness [5]. Utilization of union level facilities (facility within the study cluster) for delivery has been consistently low, 2% nationally (BDHS 2014).

**Fig. 4:**
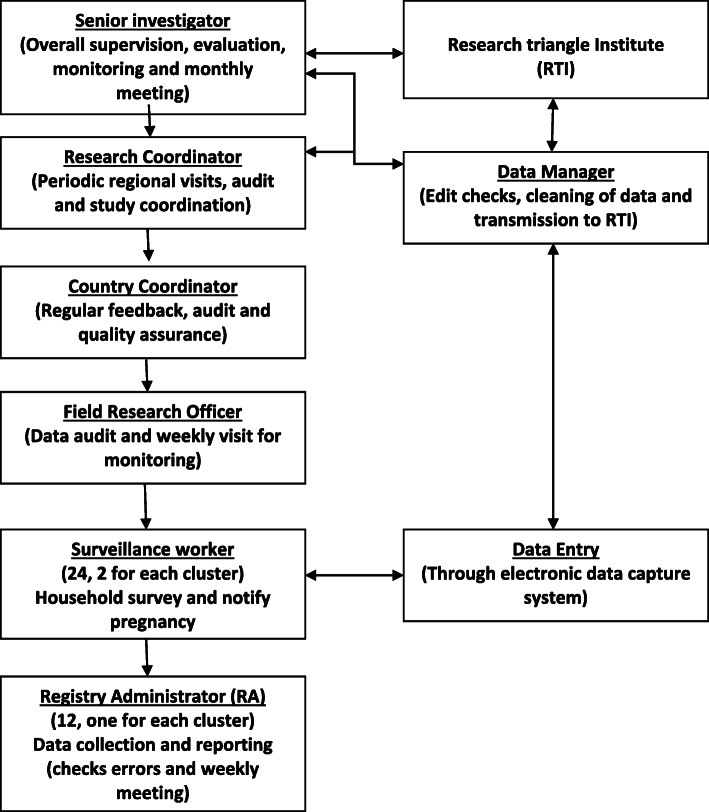
Overall administrative structure of the MNHR in Bangladesh site

A team of 12 RAs (one for each cluster) are engaged for enrollment and follow-up of the study participants under the supervision of a registry manager. Upon receiving notification of a “confirmed pregnancy” by a surveillance worker (electronically), the RA assigned for the respective cluster visits the household and invites the woman to participate in the study. After receiving written consent, the RA enrolls the pregnant woman in the study (preferably in the first trimester) and conducts antenatal and post-partum follow-up visits specified in Fig. 1. For pregnant women below 18 years of age, consent from the legal guardian and assent from the pregnant woman is obtained. If enrolled women are found absent on the scheduled follow-up visit dates during pregnancy, two planned subsequent attempts are made to collect the information. In the case of post-birth visits, if a woman leaves the study area to deliver, the follow up visit will be made upon her return. In case of natal movement for delivery within the study area, the registry administrator from the respective area conducts post-birth follow-up visits. In case of maternal, newborn or perinatal deaths in the registry, cause of death interview is conducted by project research physician preferably within a month of notification. Information collected during each of the follow-up visits is illustrated in Fig. 1. Registry enrollment and follow-up visit data collection are conducted by an electronic data capturing system developed for the Android hand-held tablet computers. RAs are supervised by a field research supervisor. Before starting enrollment, the registry administration team was trained on the consenting process, interviewing skills, anthropometric measurements, data collection tools, use of the tablets and data collection application software and cultural aspects to consider during data collection. Fortnightly meetings are organized for troubleshooting and feedback. Figure 4 shows the overall administrative structure of the MNHR for Bangladesh.

### Electronic data capture system for MNH

**Fig. 5:**
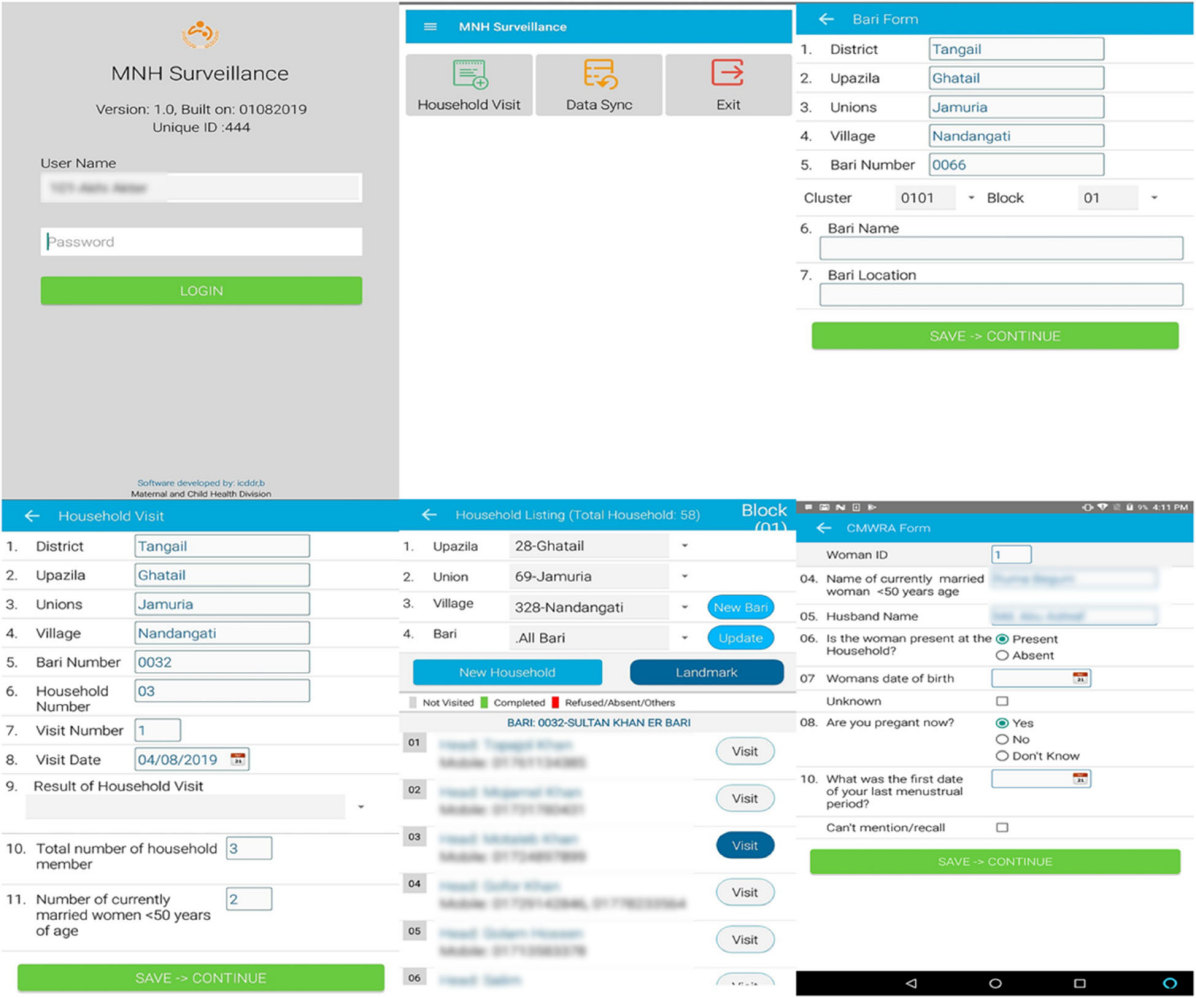
Data collection screen of surveillance application on android tablets

**Fig. 6:**
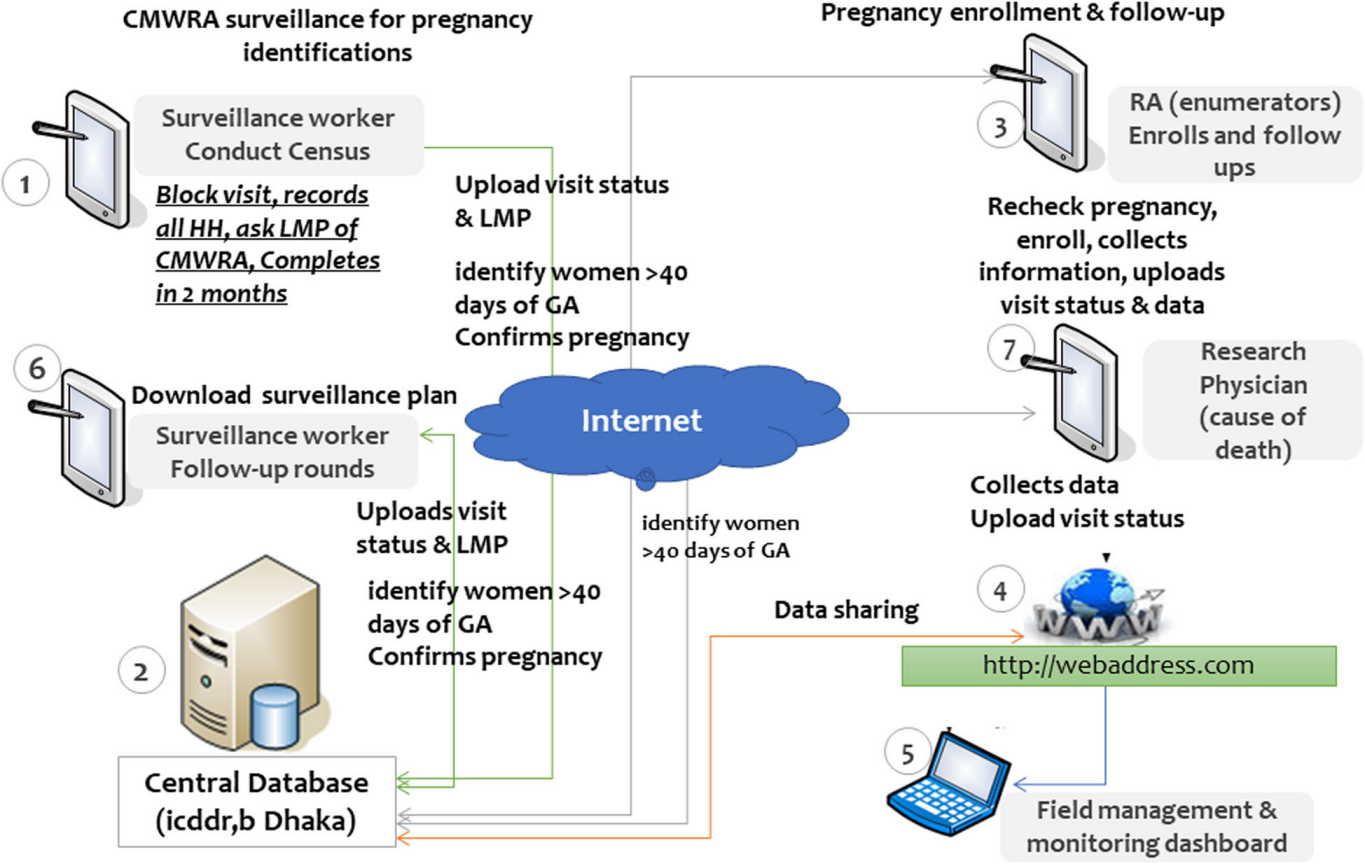
Data flow architecture of Bangladesh MNH registry site

A comprehensive automated registry data capture system has been developed through a web linked tablet-PC-based system. The system allows for married women surveillance, pregnancy enrollment and data collection during follow-up visits. The data capture system runs on the Android Platform. We are using Tablets to collect the data. Open source programming languages were used to develop the software. Figure 5 illustrates the electronic data capturing systems. Surveillance workers use a custom designed application for conducting two-monthly surveillance visits. The application prompts the surveillance workers with the visits schedules and directs the information to be collected with necessary skips and mandatory entry. The list of pregnant women identified by married women surveillance is transferred to a central server and is then transmitted to the registry application of the RA. Figure 6 illustrates the data flow architecture of the MNH registry system. The RAs then use the application for enrolling the participant. The application automatically generates a subsequent visit plan upon each pregnancy enrollment. Similarly, the registration of births automatically generates a plan for postpartum follow-up visits. Generally, the surveillance workers and registry administrators have access to internet connectivity. However, data collection can be done offline in case of poor internet access. Data are uploaded to a central sever as soon as the interview is finished or at the end of the day’s work (in case of low internet connectivity). The data capturing interfaces are exactly matched with the GN registry data forms. The data entry system is secured by password, with different permissions for entry and editing granted to different study personnel. Necessary data validation rules such as range check, inconsistency checks, data type check, logical check, cross-system consistency check, and data presence check are in-built within that application. The validation rules include range check, consistency check and abnormal values.

### Data monitoring

**Fig. 7:**
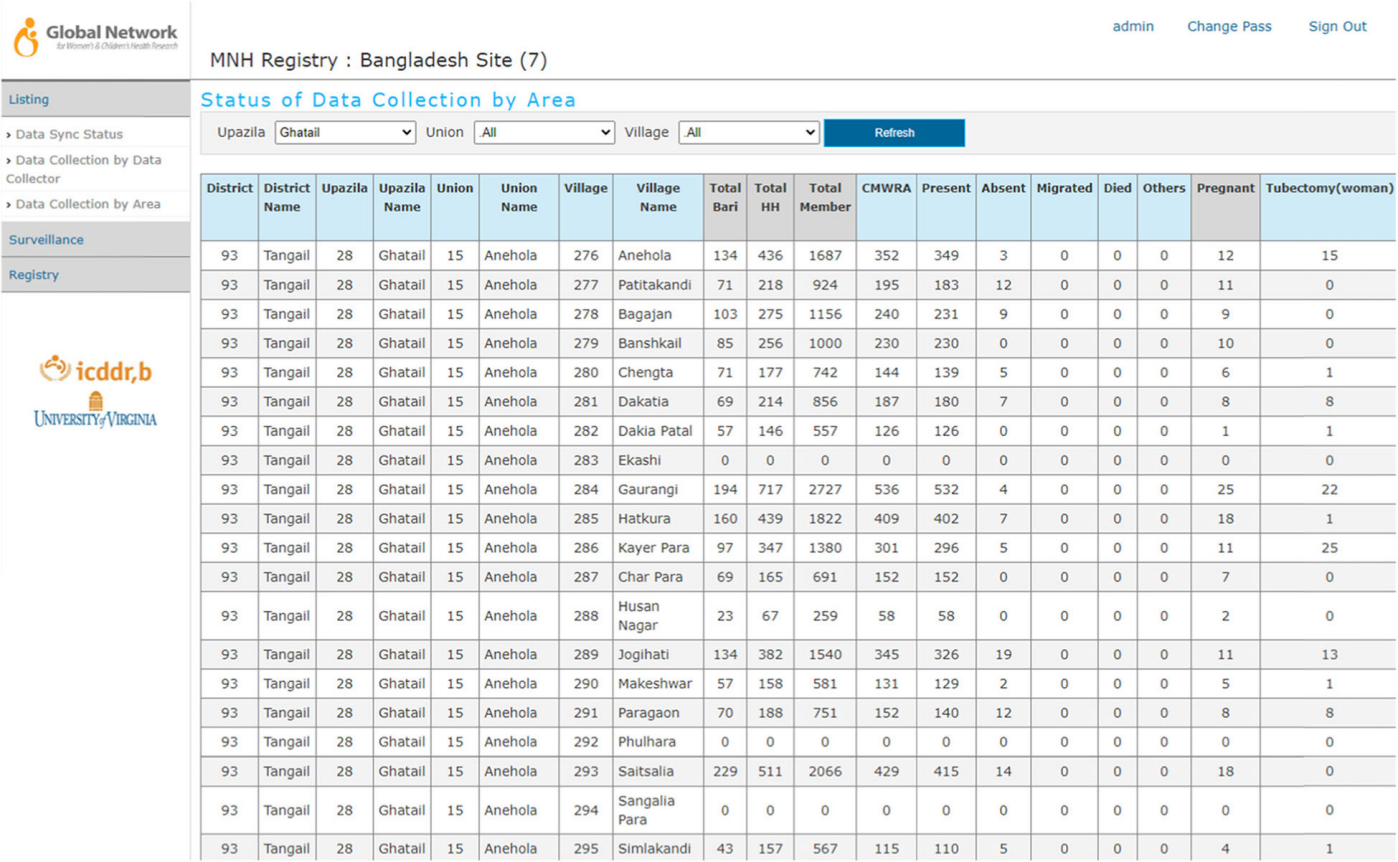
Web monitoring data dashboard

A web-linked registry dashboard, accessible to study staff on real time, has been developed to monitor activities/performance of surveillance workers and data collectors in real time by the central team as well as by field supervisors and managers. The reporting system is flexible and equipped with a query builder to generate the report in different formats. Aggregated statistics of Bari, household, members in household, number of CMWRA, and pregnant women are included. Figure 7 illustrates the monitoring dashboard.

### Data storage and backup

Data are stored locally in the tablet during data collection in the SQLite database. The Microsoft SQL Server 2008 R2 is used to store data centrally. Data is uploaded from tablets to the central server at icddr,b after completion of the day’s work. Overall, internet connectivity is not a big challenge in Bangladesh MNHR site. Poor internet connections in some pockets and low speed sometimes limit the real time data transfer. Data collected offline during poor internet connection remains stored in the tablet memory and gets uploaded as soon as the internet connection is restored. The central server uses a RAID6 hard disk to minimize the risk of data loss and server downtime. A data backup mechanism scheduled with SQL Database has been developed for automatic data backup on daily basis. Daily backups use a different hard drive and are also copied to another server in a different location. The data manager performs a manual backup of the database on a weekly basis.

## Results

In the Bangladesh MNH registry site, the initial mapping and listing of households and CMWRAs was completed during March–May 2019. In 12 study clusters, a total of 56,064 households located in 16,840 *baris* were listed (Table 4). After the initial round of surveillance, the registry was comprised of 221,462 people ranging from 17,891 to 19,330 in each cluster. CMWRA were found in 88% of the households. We found some variability in availability of CMWRA among the clusters, ranging from 84% in Jamuria cluster to as high as 92% in Elenga. A total of 49,269 CMWRA were listed with 2% absenteeism in the initial round.

**Table 4:** Demography of Bangladesh MNH registry site clusters

Cluster	Total Bari listed (#)	Total households listed (#)	Total population (#)	Currently Married Women in Reproductive Age (CMWRA)	Average Availability of CMWRA in HHs	Women less susceptible to become pregnant (#)	CMWRA reported currently pregnant (%)
**Anehola**	1667	4854	19,308	4102	85%	551	4%
**Digal-kandi**	967	4589	18,034	3917	85%	620	5%
**Jamuria**	1390	4506	17,891	3774	84%	659	5%
**Bangra**	1049	4567	18,114	4001	88%	604	5%
**Bir Basunda**	1908	4723	18,335	4265	90%	297	5%
**Elenga**	944	4431	17,937	4067	92%	702	4%
**Kok Dahara**	1979	4973	19,330	4523	91%	501	5%
**Nagbari**	1574	4690	17,936	4254	91%	337	4%
**Narandia**	1703	4558	18,538	3857	85%	502	4%
**Paikara**	1180	4858	18,445	4410	91%	521	5%
**Salla**	1144	4591	18,508	4056	88%	716	5%
**Sahadeb-pur**	1335	4724	19,086	4043	86%	534	5%
**Total**	16,840	56,064	221,462	49,269	88%	6544	5%

**Fig. 8:**
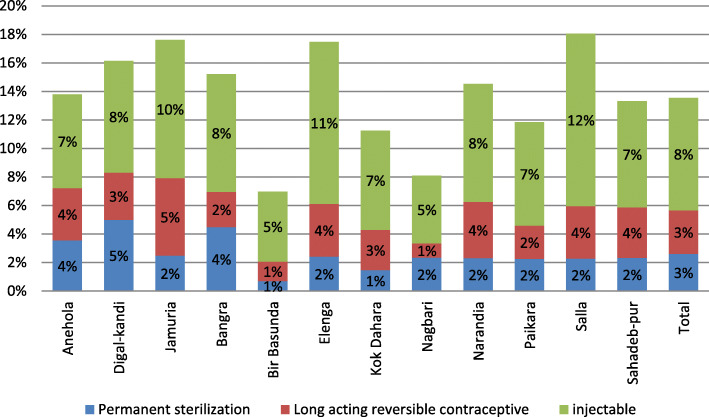
Prevalence of permanent sterilization, long-acting reversible contraceptives and injectable contraceptive by clusters

Of the total CMWRAs, 6544 (13%) were less susceptible to becoming pregnant as they were either permanently sterilized or on long-acting or regular injectable contraceptives. The year 1 CMWRA surveillance continued with 42,725 listed women after excluding the women who were not/less susceptible to pregnancy. Large variability was observed in contraceptive use (Fig. 8). Coverage of permanent sterilization (3%) and long acting methods (3%) were much lower compared to injectable contraception (8%). Prevalence of the three selected contraceptives was lower in Bir Basunda (7%) and Nagbari (8%) clusters, while higher prevalence was found in Salla, Elenga and Jamuria (~ 18%). Overall, 5% of the listed CMWRAs were reported as currently pregnant with no large variations across the clusters (Table 4).

## Discussion

In LMIC, where vital registration is not well established with universal coverage, alternative registration systems (like Demographic Health Survey, population census) have been found to be important data source [3]. MNHR has advantages over available alternative registration systems as it uses the same indicator and standard definitions across all sites. Moreover, data is collected continuously in real-time rather than periodic surveys [3]. Bangladesh site is also implementing a full electronic data capturing system (EDC). EDC has some proven benefits in limiting data collection errors, data quality checks, efficiency, real-time data collection progress monitoring, and data sharing in comparison to paper-pen data capturing [6]. Like other GN sites, Bangladesh MNHR site will also continuously generate critical maternal and newborn health outcome estimates. Information on maternal background and health characteristics, as well as exposure to essential interventions, will be used to identify the predisposing predictors for maternal and newborn outcomes, which will guide to identify critical interventions and open windows of opportunity.

Implementation of EDC in rural and resource-limited areas depends on the availability of technical infrastructure, additional training of field staff, higher upfront costs to purchase equipment and develop software, acceptance of study participants, and additional data security measures [7]. Like other GN sites, Bangladesh site has deployed a team of a professional programmer to develop the electronic data capturing systems and troubleshooting. Candidates went through strict scrutiny during the recruitment of local women as surveillance workers and registry administrators. Intensive start-up training and quarterly refreshers for surveillance workers, registry administrators and supervisors were necessary to improve competency and standardization of the field teams. Three tiers of field supervisors ensured both performance and quality monitoring of pregnancy identification and enrollment in the registry. Like other similar registry systems, the establishment of the Bangladesh MNHR systems required high upfront cost and substantial direct engagement of study investigators, information technology specialists, data managers, and GIS specialists.

Experience from our full automated registry site would generate critical learning for the growing interest in establishing national electronic health records and digitalization of health information systems. Bangladesh is already in the expansion phase of digital health information systems including individual health records using DHIS2 platform (Directorate General of Health Services) [8] and a locally developed custom-designed e-MIS tool (Directorate General of Family Planning) [9]. Our experience of pregnancy registration, intervention coverage, and outcome tracking would provide important contextualized considerations for both design and implementation of individual-level health information capturing and sharing systems.

We anticipate completing follow-up of ~ 3000 birth outcomes in year 1 and generating the first round of outcome estimates from Bangladesh site by June 2020.

## Conclusion

EDC has advantages of less error-prone data collection. In the EDC system, data directly upload in the data management server which minimizes the time for data entry and avoids error during data entry. Real-time data collection progress and data quality monitoring are possible in the EDC system through web-based dashboard. However, the implementation of EDC in a resource-poor setting depends on technical infrastructure, skilled staff, software development, stable internet connection, community acceptance, and a data security system. Our experience of pregnancy registration, intervention coverage, and outcome tracking would provide important contextualized considerations for both the design and implementation of individual-level health information capturing and sharing systems.

## Data Availability

Data from the study will be available at the NICHD data repository (N-DASH): https://dash.nichd.nih.gov/.

## Abbreviations

MNH: Maternal and newborn health
Global Network, GN: The Global Network for Women’s and Children’s Health Research
MNHR: Maternal Newborn Health Registry
CMWRA: Currently married women in reproductive age
EDC: Electronic data capturing system
LMICs: Low and middle income countries
COD: Cause of death
CBR: Crude birth rate
UH&FWC: Union Health and Family Welfare Centers
GIS: Geographic information system
ODK: Open Data Kit
RMSE: Root Mean Square Error
FS: Field Supervisors
